# Soil Inoculation With Beneficial Microbes Buffers Negative Drought Effects on Biomass, Nutrients, and Water Relations of Common Myrtle

**DOI:** 10.3389/fpls.2022.892826

**Published:** 2022-05-27

**Authors:** Soghra Azizi, Masoud Tabari, Ali Reza Fallah Nosrat Abad, Christian Ammer, Lucia Guidi, Martin K.-F. Bader

**Affiliations:** ^1^Faculty of Natural Resources, Tarbiat Modares University, Tehran, Iran; ^2^Department of Forestry, Faculty of Natural Resources and Marine Sciences, Tarbiat Modares University, Tehran, Iran; ^3^Soil and Water Research Institute, Agricultural Research Education and Extension Organization (AREEO), Karaj, Iran; ^4^Silviculture and Forest Ecology of the Temperate Zones, Georg-August-Universität Göttingen, Göttingen, Germany; ^5^Department of Agriculture, Food and Environment, University of Pisa, Pisa, Italy; ^6^Department of Forestry and Wood Technology, Linnaeus University, Växjö, Sweden

**Keywords:** arbuscular mycorrhizal fungi, plant growth-promoting rhizobacteria, water deficit stress, *Myrtus communis*, drought

## Abstract

Common myrtle (*Myrtus communis* L.) occurs in (semi-)arid areas of the Palearctic region where climate change, over-exploitation, and habitat destruction imperil its existence. The evergreen shrub is of great economic and ecological importance due to its pharmaceutical value, ornamental use, and its role in urban greening and habitat restoration initiatives. Under greenhouse conditions, we investigated the effect of soil inoculation with arbuscular mycorrhizal fungi (AMF) and plant growth-promoting rhizobacteria (PGPR) on biomass allocation, water relations, and nutritional status of drought-stressed myrtle seedlings. Single and dual AMF (*Funneliformis mosseae* and *Rhizophagus irregularis*) and PGPR (*Pseudomonas fluorescens* and *P. putida*) soil inoculations were applied to myrtle seedlings growing under different soil water regimes (100, 60, and 30% of field capacity) for 6 months using a full factorial, completely randomized design. AMF and PGPR treatments, especially dual inoculations, alleviated negative drought effects on biomass and morpho-physiological traits, except for water-use efficiency, which peaked under severe drought conditions. Under the greatest soil water deficit, dual inoculations promoted leaf biomass (104%–108%), root biomass (56%–73%), mesophyll conductance (58%), and relative water content (1.4-fold) compared to non-inoculated controls. Particularly, dual AMF and PGPR inoculations stimulated nutrient dynamics in roots (N: 138%–151%, P: 176%–181%, K: 112%–114%, Ca: 124%–136%, and Mg: 130%–140%) and leaves (N: 101%–107%, P: 143%–149%, K: 83%–84%, Ca: 98%–107%, and Mg: 102%–106%). Our findings highlight soil inoculations with beneficial microbes as a cost-effective way to produce highly drought resistant seedling stock which is vital for restoring natural myrtle habitats and for future-proofing myrtle crop systems.

## Introduction

Drought is one of the most important environmental stresses limiting growth and metabolic processes in plants (Trenberth et al., 2014). Plant responses to drought depend on the severity and duration of the drought period and on the stage of plant development. Severe drought stress impairs physiological processes, inhibits growth, and may eventually lead to plant death (Anjum et al., 2011). Drought-related reductions in root water uptake hamper tissue hydration thereby increasing osmotic stress (Aroca et al., 2008) and decreasing leaf water potential, foliage quantity, and dry mass so that at each developmental stage nutrient uptake and transfer are reduced (Anjum et al., 2011). As a result of the drought-induced deterioration of plant water status, membrane permeability and transport processes decline and the reduced mass flow of nutrients affects root uptake and nutrient allocation in turn (Sardans et al., 2008).

Soil inoculation with arbuscular mycorrhizal fungi (AMF) and plant growth-promoting rhizobacteria (PGPR) is currently being widely explored as a new approach to alleviate detrimental drought effects and to enhance crop drought resistance in agriculture and horticulture in arid and semi-arid regions. However, its potential for sustainable arboriculture and restoration remains virtually unexplored. As keystone soil engineers, AMF can partially offset the negative effects of drought stress on plants by increasing root water and nutrient uptake (especially recalcitrant nutrients, such as P, Zn, and Cu), photosynthetic activity, the production of antioxidant enzymes, and not least because of their beneficial effects on the rhizosphere environment including the rhizomicrobiome (Kung’u et al., 2008; Bárzana et al., 2015; Yin et al., 2016). In particular, AMF improve soil structural quality through the formation of stable soil aggregates supported by hyphal exudates and the enmeshment of particles in mycelial networks resulting in enhanced nutrient and water availability as well as reduced soil erosion (Curaqueo et al., 2010; Mardhiah et al., 2016). The widespread AMF species *Funneliformis mosseae* and *Rhizophagus irregularis* occur in various habitats and have a vast host range making them prime candidates for crop yield and quality improvements under drought (Ortiz et al., 2015; Zhang et al., 2019). The genus *Pseudomonas* contains the most important growth-promoting bacteria that regulate the amount of ethylene through production of siderophores, plant hormones, synthesis of antibiotics, phosphorus absorption, nitrogen fixation, and synthesis of enzymes (Henry et al., 2008).

Investigations on the drought mitigation potential of soil microorganisms often show improvements in growth, physiological, and biochemical traits along with a higher uptake of water and mineral elements in inoculated plants (e.g., Ortiz et al., 2015; Ullah et al., 2016). The literature is heavily crop-dominated but the few studies on woody plants (mainly horticultural species) also suggest better water supply evidenced by favourabale plant water relations, enhanced macro- and micronutrient uptake, greater growth, larger osmoregulant pools, and an upregulation of the antioxidant defence in AMF- and/or PGPR-inoculated individuals under drought (Abbaspour et al., 2012; Danielsen and Polle, 2014; Zarik et al., 2016; Zhang et al., 2019).

The medicinal plant *Myrtus communis* L. is an evergreen shrub (<3 m tall) of the Myrtaceae family. It is native to southern Europe and western Asia and grows well in sub-Mediterranean climates (Sumbul et al., 2011) including some provinces of Iran. *Myrtus communis* has many pharmacological properties and contains active components, including essential oils such as depantine or myrtenol, and is therefore of economic interest to the pharmaceutical industry (Moghrani and Maachi, 2008). Habitat destruction and over-exploitation have pushed *M. communis* to the brink of extinction in its natural habitats in Iran and other regions of the world, where it used to form dense, extensive stands in the past (Amiri et al., 2015; González-Varo et al., 2015). Besides its widespread cultivation for ornamental and medicinal reasons, myrtle is well-suited for the rehabilitation of degraded lands and the development of parks and (sub-)urban green spaces in arid areas (Azizi et al., 2021).

To better manage current and future shortages of water resources projected under climate change scenarios, research aimed at improving myrtle drought resistance is urgently needed.

The present study is part of a larger, overarching project on myrtle responses to drought. In a complementary trial, using the same experimental setup, we previously investigated mycorrhizal colonization, seedling survival, growth, and leaf gas-exchange along with oxidative damage and antioxidant defense (Azizi et al., 2021). AMF and PGPR soil inoculation treatments increased seedling survival under drought. Especially the dual AMF and PGPR inoculations had a positive impact on foliar physiology and led to a substantial decrease in oxidative damage as evidenced by reduced levels of oxidative stress markers, less electrolyte leakage, and fewer pigment losses in drought-exposed seedlings. Soil microbial-driven increases in the pool size of enzymatic and non-enzymatic antioxidants, including essential oils, indicated an upregulation of the antioxidant defense system of the seedlings subjected to drought (Azizi et al., 2021).

In the present study, we tested whether soil inoculation with beneficial microorganisms provides a viable option for adaptive management of myrtle propagation for cultivation and reforestation. We investigated the potential of single and dual AMF and PGPR inoculation for mitigating drought effects on biomass, leaf water relations as well as root and foliar nutrient content of common myrtle. We hypothesized that AMF and PGPR inoculation will improve biomass production, water relations and nutrient status in leaves and roots of *M. communis* seedlings under drought. We further anticipated a stronger efficacy of dual vs. single microbial inoculations in terms of mitigating the detrimental effects of drought on productivity and physiological functioning.

## Materials and Methods

### Experimental Design

In early July 2017, 2-year-old potted seedlings (6 ± 1 mm in root collar diameter, 37 ± 3 cm in shoot length) of *M. communis* (originated from seed) raised in the greenhouse of Faculty of Agriculture, Tarbiat Modares University, Iran (35°44′ N, 51°10′ E, and 1,215 m a. s. l.), were transferred to 5-L plastic pots containing a mixed soil (agricultural soil, green manure + sand + coco peat in a ratio of 1: 1: 1: 2) with physico-chemical properties indicated in Table 1. The green manure consisted of decomposed plant litter originating from various local forest trees, mainly oak and hawthorn species (*Quercus* spp., *Crataegus* spp.) and pistachio (*Pistacia vera*).

**Table 1:** Physico-chemical properties of the soil used for growing *Myrtus communis* seedlings.

Texture	Sand (%)	Clay (%)	Silt (%)	Bulk density (g cm^−3^)	FC (m^3^ m^−3^)	PWP (m^3^ m^−3^)	EC (dS m^−1^)	pH
Clay-loam	33	29	38	1.59	0.34	0.13	1.61	7.9
**N (%)**	**P (mg kg^−1^)**	**K (mg kg^−1^)**	**OC (%)**	**OM (%)**	**Fe (mg kg^−1^)**	**Zn (mg kg^−1^)**	**Mn (mg kg^−1^)**	
0.18	5.6	458	1.77	3.06	9.7	3.36	10	

PWP, permanent wilting point; EC, electrical conductivity; OC, organic carbon content; and OM, organic matter content.

The experiment was carried out as a 3 × 7 factorial (water regime × microorganism inoculation) in a completely randomized design with three replicates each comprising four seedlings. The following microorganism inoculation treatments were applied: (1) control (no inoculation), (2) AMF-inoculation with *Funneliformis mosseae*, (3) AMF-inoculation with *Rhizophagus irregularis*, (4) combined inoculation of *F. mosseae* + *R. irregularis*, (5) PGPR-inoculation with *Pseudomonas fluorescens*, (6) PGPR-inoculation with *Pseudomonas putida*, and (7) combined inoculation of *P. fluorescens* + *P. putida*. For mycorrhizal fungi 40 g of inoculum (100 propagules g^−1^ of carrier material) and for *Pseudomonas* bacteria 15 ml of inoculum containing 10^7^ ml^−1^ live bacterial cell were used. For the dual AMF inoculation, 40 g of each fungus was used and for the dual PGPR treatment, 15 ml of each bacterium were applied. Inocula were prepared from the Microbial Bank of the Microbiology Department of the Soil and Water Research Institute of Tehran, Iran. Water deficit stress included 100% field capacity (FC100, no stress), 60% FC (FC60, mild stress), and 30% FC (FC30, severe stress). Drought stress was applied *via* the watering regime following Zarik et al. (2016) for 180 days.

### Root Mycorrhizal Colonization

The percentage of AMF-colonized roots was assessed in a complementary study (Azizi et al., 2021). Mycorrhizal colonization in control plants was highest in the FC100 treatment (9.4%) and dropped by nearly 80% under FC30 conditions. By contrast, mycorrhization in singly and dually inoculated plants ranged from 51 to 72.7% under FC100 conditions and only halved in response to increasing water limitation.

### Biomass Allocation

For biomass determination, one seedling of each replicate was removed from the soil and after rinsing off the soil attached to the roots, seedlings were separated into their main organs and dried at 70°C for 48 h. Then, a digital scale with an accuracy of 0.0001 g, was used to measure leaf biomass (LB), stem biomass (SB), and root biomass (RB).

### Water Relations and Gas Exchange Parameters

At the end of the experiment, the intracellular CO_2_ concentration (*C*_i_) was measured using a portable gas exchange device LI-6400 (LiCor Inc., Lincoln, NE, United States). For this purpose, four fully developed, healthy leaves were selected from the top of the crown from each of three seedlings per treatment. Measurements were taken between 9 and 12 am on a sunny day with a light intensity of 1,400 μmol m^−2^ s^−1^ (Dong et al., 2017). To measure the relative water content (RWC), three healthy, fully developed leaves from the top of the crown from each of three seedlings per treatment were sampled. After determination of fresh weight (FW), the leaf samples were placed in distilled water in the dark for 24 h to maximize absorption for turgor weight (TW) determination. Then turgid leaves were placed in an oven at 70°C for 48 h. Leaf weight was measured after drying (DW) and RWC was calculated according to Equation (1) (Yang et al., 2007).


(1) RWC=[(FW−DW)/(TW−DW)]×100

Following the results of photosynthesis and transpiration in our previous study (Azizi et al., 2021), mesophyll conductance (*g*_m_) and water use efficiency (WUE) were determined according to Equations (2, 3) (Moradi-ghahderijani et al., 2017; Biswas et al., 2019).


(2) $$g_{m}=\frac{A}{C_{i}}$$

(3) $$\mathrm{WUE}=\frac{A}{E}$$

where, A = photosynthesis, *C*_i_ = intracellular CO_2_ concentration, and E = transpiration.

### Root and Leaf Nutrients

At the end of the experiment, a representative seedling per replicate (average height and diameter) was chosen and its root and leaves were washed and placed in an oven (at 70°C) for 48 h until dry weight was reached. For root and foliar nitrogen determination by the Kjeldahl method, 0.5 g powdered sample was weighed and transferred to digestion tubes. One catalyst tablet was added with 10 ml of concentrated sulfuric acid. The tubes were placed in a digestion furnace for 3–4 h at 40°C (until the color of the samples turned blue). After cooling, 10 ml of distilled water was added to each of the tubes prior to titration (Chapman and Pratt, 1962).

To measure phosphorus content a calorimetric method (yellow color of molybdate and vanadate) was utilized. After digestion of 1 g of powdered leaf and root sample, 5 ml of extract was poured into a 25 ml balloon and 5 ml of ammonium molybdate vanadate solution was added. Then, absorbance at 480 nm was read using a spectrophotometer (Lambda 45-UV/Visible, PerkinElmer, Waltham, MA, United States; Chapman and Pratt, 1962).

To measure the concentration of K, Ca, and Mg of roots and leaves, the samples were dried in an oven at 48°C for 48 h (Ribeiro et al., 2002). Then, 1 g of dried and powdered sample material was mixed with 10 ml of concentrated nitric acid and after staying under the hood for 12 h, it was exposed to 80°C for 2 h. The prepared solution after mixing with 3 ml of concentrated perchloric acid was kept at 160°C for 5 h. After cooling, the prepared samples were smoothed with filter paper and reached the 25 ml volume. Then, the content of the above elements was assessed using an atomic absorption spectrometer (Ribeiro et al., 2002).

### Statistical Analysis

The data were analyzed using SPSS statistical software version 23 (SPSS Inc., Chicago, IL, United States), and graphs were drawn in Excel software version 2016 (Microsoft Office, 2016). Kolmogorov–Smirnov and Levene tests were used to evaluate the normality and variance homogeneity of the data, respectively. Two-way ANOVA was used to determine the overall significance of the treatments, and Duncan’s new multiple range test was used as a *post hoc* test to compare differences between group means.

## Results

The two-way ANOVA revealed a significant water regime × microbial inoculation effect on organ-specific biomass, mesophyll conductance, relative water content, and root P and leaf Mg contents. All variables that remained unaffected by the two-way interaction were significantly influenced by the two main effects, apart from water use efficiency, which only showed a significant response to the water regime (Table 2).

**Table 2:** Results of a two-way ANOVA testing the effect of microorganism inoculation on biomass allocation, water relations, and elemental concentration of *M. communis* seedlings under three different water regimes (100, 60, and 30% of field capacity).

	Water regime		Inoculation		Water regime × inoculation	
Traits	*df*	*F* value	*df*	*F* value	*df*	*F* value
Leaf biomass	2	150.05^***^	6	19.87^***^	12	2.05^*^
Stem biomass	2	92.32^***^	6	10.73^***^	12	0.585^ns^
Root biomass	2	365.78^***^	6	30.29^***^	12	3.33^**^
Intracellular CO_2_ concentration	2	618.80^***^	6	18.21^***^	12	0.446^ns^
Mesophyll conductance	2	815.02^***^	6	33.34^***^	12	6.44^**^
Water use efficiency	2	4.84^*^	6	0.316^ns^	12	0.176^ns^
Relative water content	2	456.07^***^	6	14.21^***^	12	2.19^*^
N root	2	733.15^***^	6	25.28^***^	12	0.189^ns^
P root	2	1578.17^***^	6	35.15^***^	12	2.06^*^
K root	2	1403.53^***^	6	27.88^***^	12	0.837^ns^
Ca root	2	409.95^***^	6	11.75^***^	12	0.655^ns^
Mg root	2	698.02^***^	6	15.03^***^	12	0.823^ns^
N leaf	2	671.28^***^	6	19.59^***^	12	1.39^ns^
P leaf	2	2642.19^***^	6	51.48^***^	12	1.38^ns^
K leaf	2	2126.72^***^	6	60.42^***^	12	1.47^ns^
Ca leaf	2	597.77^***^	6	16.55^***^	12	0.26^ns^
Mg leaf	2	504.173^***^	6	16.70^***^	12	2.34^*^

ns No significant difference.

* *p* ≤ 0.05;

** *p* ≤ 0.01;

*** *p* ≤ 0.001.

### Biomass Allocation

**Figure 1:**
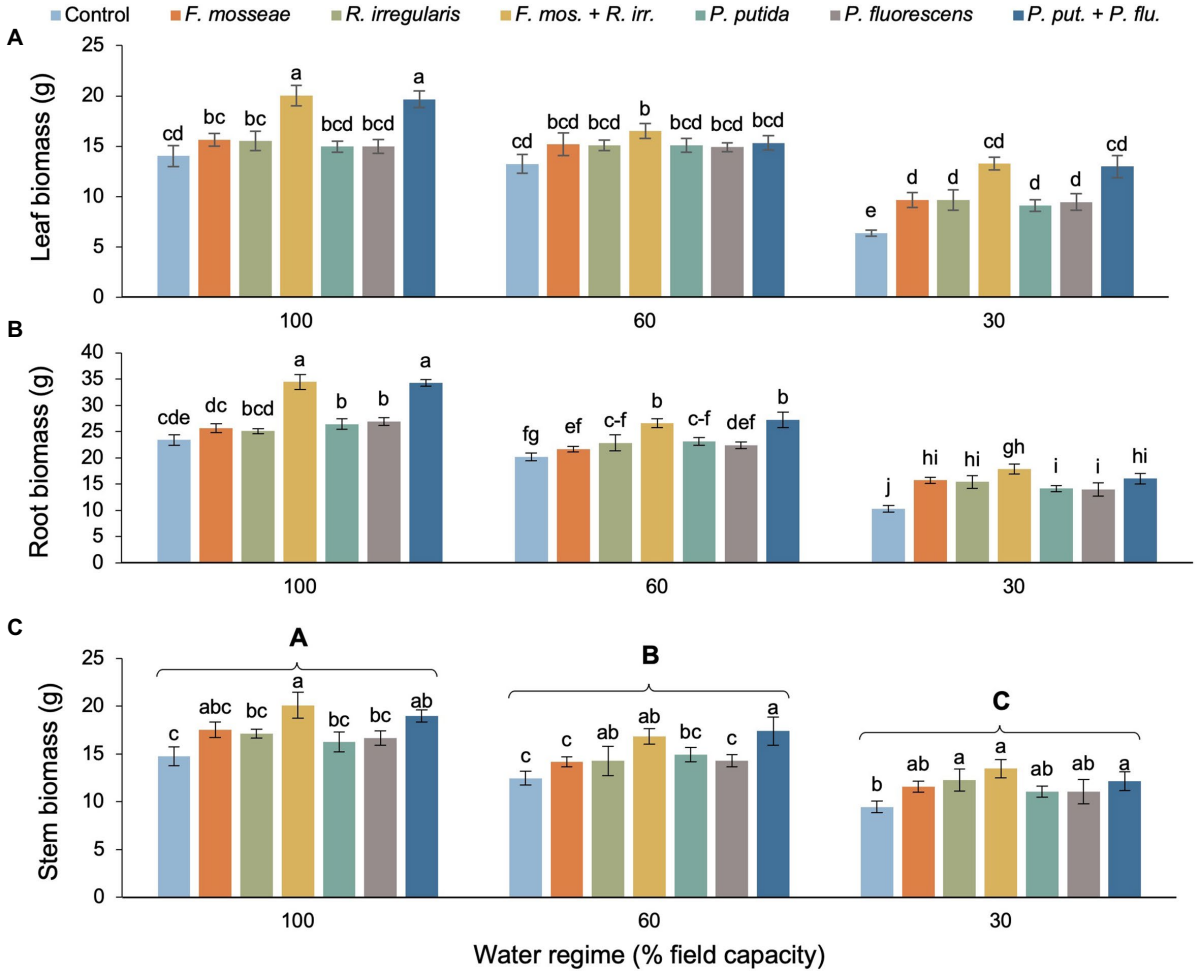
Effect of water regime and soil microbial inoculation on the biomass allocation of *Myrtus communis* seedlings. For leaf biomass and root biomass there was a significant water regime × inoculation treatment interaction (Table 2), hence different lower-case letters indicate statistically significant differences across the 21 treatment combinations. For stem biomass, only the main effects of water regime and inoculation treatment were significant (Table 2), thus upper-case letters indicate a significant difference between different levels of drought stress and lower-case letters indicate a significant difference between different inoculation treatments within a water regime (Duncan’s new multiple range test, *α* = 0.05).

The detrimental effects of drought were evident in dry mass of all organs (Table 2). However, single AMF and PGPR inoculations (partly) compensated and dual inoculations sometimes even overcompensated the negative effects of drought stress compared to the well-watered, non-inoculated control (Figures 1A–C). For instance, in the FC60 treatment, dual inoculation almost always resulted in significantly larger biomass accrual than both the FC60 and the FC100 non-inoculated control. The strongest inoculation effects occurred in the FC30 treatment of leaf biomass, where dual AMF and PGPR more than doubled the values seen in the control (Figure 1A). The stimulation under severe drought was less pronounced in roots, where dual AMF inoculation produced 73% higher biomass than the control, but this was not statistically different from the magnitude seen in single AMF inoculations. Similarly, the dual PGPR inoculation stimulated root biomass significantly by 56%, but this was not significantly different from the increase related to single PGPR inoculations (Figure 1B). Under severe drought, only the single *R. irregularis* inoculation, the dual AMF and the dual PGPR inoculation caused significant increases in stem biomass compared to the control. The stem biomass values linked to the remaining inoculation treatments were in between the values of the control and the three effective inoculation treatments and therefore did not differ significantly from either (Figure 1C).

### Water Relations

**Figure 2:**
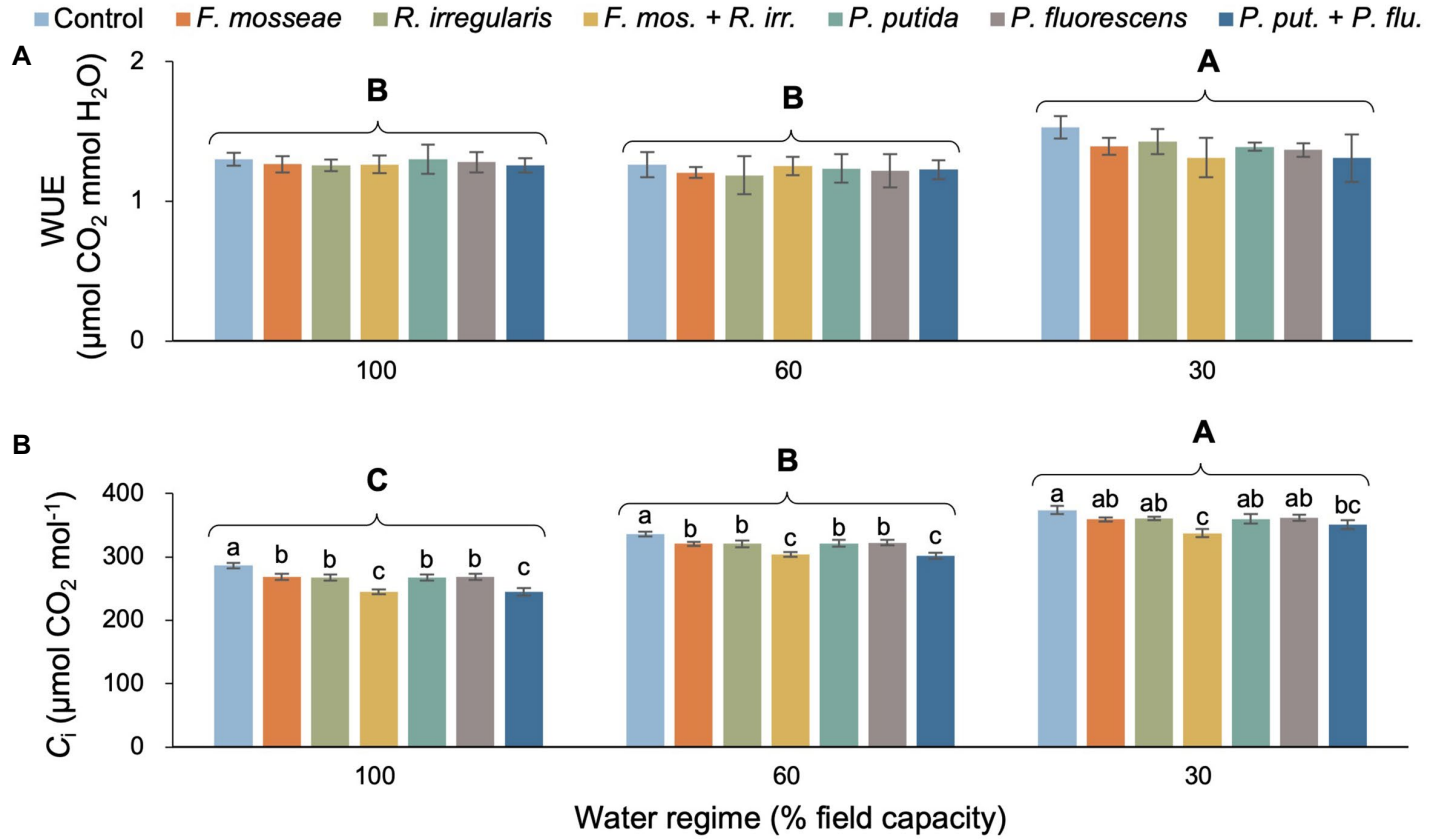
Effect of water regime and soil microbial inoculation on the water use efficiency (WUE; **A**) and intracellular CO_2_ concentration (*C_i_*; **B**) of *M. communis* seedlings. For both variables, only the main effects of water regime and inoculation treatment were significant (Table 2). Different upper-case letters indicate significant differences between water regimes. Different lower-case letters indicate significant differences between microbial inoculation treatments within each water regime (Duncan’s new multiple range test, *α* = 0.05).

There was neither a statistically significant two-way interaction nor a microbial inoculation effect on seedling WUE (Table 2; Figure 2A). However, the severe drought treatment (FC30) produced significantly higher WUE compared to the remaining watering regimes, which showed similar values (Figure 2A). We did not detect a significant water regime × microbial inoculation interaction but both main effects were statistically significant (Table 2). Irrespective of the inoculation treatment, the intracellular CO_2_ concentration (*C*_i_) increased significantly with increasing drought stress (Figure 2B). Regardless of the water regime, the dual AMF inoculation induced significantly lower *C*_i_ values than the control and single inoculations (Figure 2B). In the FC30 treatment, the dual AMF treatment caused an 11% decrease in *C*_i_ compared to non-inoculated control seedlings (Figure 2B). A similar pattern was seen for the dual PGPR inoculation treatment across drought conditions, except for the FC30 group, where seedlings dually inoculated with PGPR showed *C*_i_ values that were significantly lower than the control but similar to all singly inoculated seedlings (Figure 2B).

**Figure 3:**
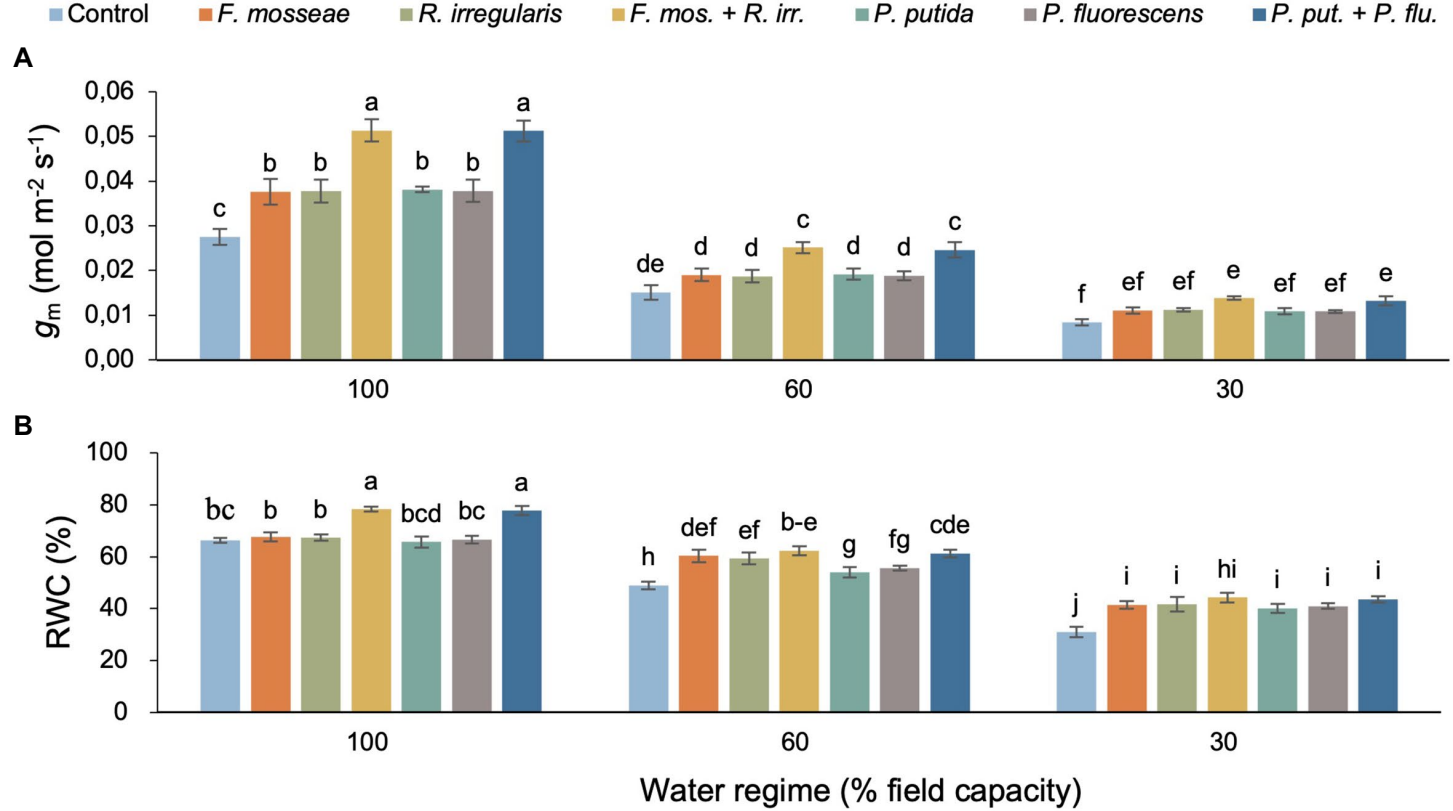
The interaction effect of water regime and soil microbial inoculation on the mesophyll conductance (*g*_m_; **A**) and relative water content (RWC; **B**) of *M. communis* seedlings. For both variables, there was a significant water regime × inoculation treatment interaction. Different lower-case letters thus indicate significant differences between microbial inoculation treatments across water regimes (Duncan’s new multiple range test, *α* = 0.05).

Mesophyll conductance (*g*_m_) declined with increasing soil water deficit, but this effect varied with inoculation treatment resulting in a significant two-way interaction (Table 2; Figure 3A). Dual AMF and PGPR inoculation caused the highest *g*_m_ values in well-irrigated (+86% relative to the control) and mildly drought-stressed seedlings (+63%–66% relative to the control; Figure 3A). Under severe water deficit, dual AMF and PGPR inoculation increased *g*_m_ by 64 and 57%, respectively, compared to the non-inoculated control (Figure 3A).

Water regime and microbial inoculation also had a significant interactive effect on RWC (Table 2). Dual AMF and PGPR inoculation significantly promoted leaf RWC in well-irrigated seedlings while single inoculations had no effect (Figure 3B). Under FC60 conditions, all AMF inoculations equally improved RWC relative to the control (>20%) and a similar increase was seen in the dual PGPR treatment (Figure 3B). The effects of the single PGPR inoculations were slightly less pronounced but still represented a significant improvement compared to the control (Figure 3B). Under severe drought (FC30), all microbial inoculations significantly increased foliar RWC between 30 and 43% without significant differences among inoculation treatments (Figure 3B).

### Root Nutrient Concentration

**Figure 4:**
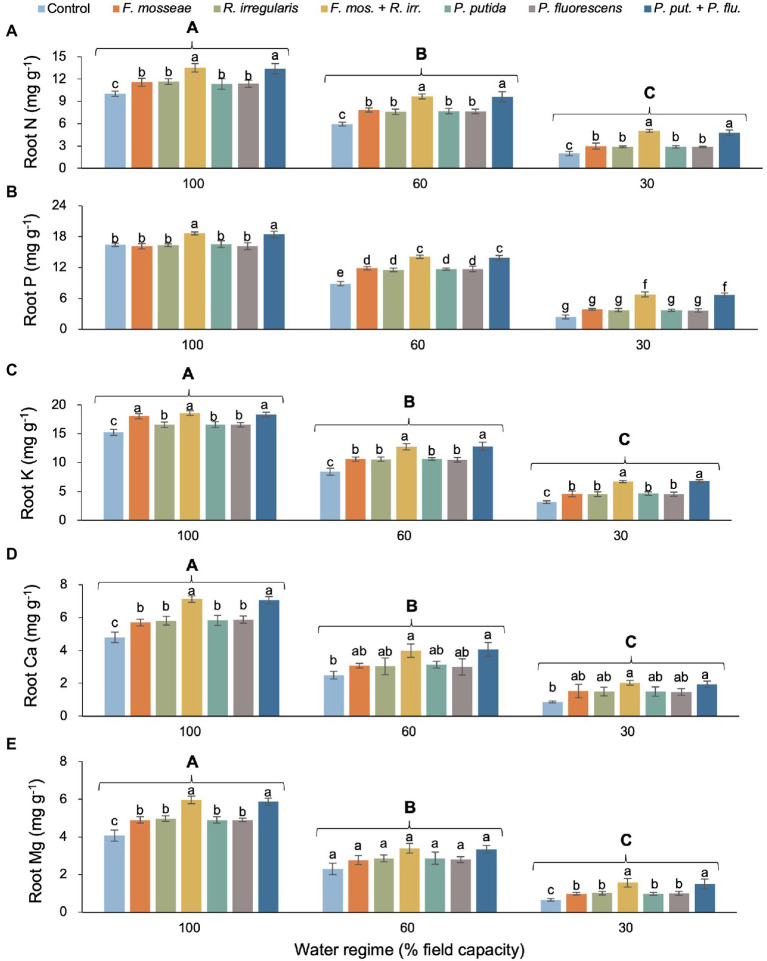
Effect of water regime and soil microbial inoculation on the root N **(A)**, P **(B)**, K **(C)**, Ca **(D)**, and Mg **(E)** of *M. communis* seedlings (expressed on a dry weight basis). A significant water regime × inoculation treatment interaction only occurred for root P, while for all other root nutrients only the main effects were statistically significant. Accordingly, different lower-case letters indicate significant differences between microbial inoculation treatments across levels of water deficit for root P but only within water regimes in the remaining root nutrients (Duncan’s new multiple range test, *α* = 0.05). Different upper-case letters indicate significant differences between water regimes (Duncan’s new multiple range test, *α* = 0.05).

Except for P, there was no significant interaction between water regime and microbial inoculation for any of the tested root nutrients but both main effects were statistically significant (Table 2). With increasing drought stress, root N, P, K, Ca, and Mg content decreased, but the addition of AMF and PGPR, especially the dual inoculations, significantly improved the nutrient status of seedling roots (Figures 4A–E). Across inoculation treatments, root N declined by 32% in mildly drought-stressed seedlings and by 72% under severe drought compared to well-watered control conditions (Figure 4A). Across water regimes, we observed the same inoculation-induced stimulatory pattern: all single AMF and PGPR inoculations caused similar increases in root N relative to the non-inoculated control (FC100: *ca.* 16%, FC60: *ca.* 30%, and FC30: *ca.* 45%), and the dual inoculations had even larger effects of equal magnitude among AMF and PGPR (FC100: *ca.* 34%, FC60: *ca.* 62%, and FC30: *ca.* 145%; Figure 4A).

Root P also showed a general decline with increasing drought intensity, but remarkably, single AMF and PGPR inoculations failed to improve root P status over the non-inoculated control in the FC100 and FC30 treatments (Figure 4B). Only under mild drought stress (FC60), single microbial inoculations significantly promoted root P uptake by a similar magnitude in comparison to the control. However, across water regimes, dual inoculations resulted in the largest increase in root P, significantly exceeding values seen in singly inoculated and control seedlings (Figure 4B). Root K, Ca, and Mg decreased by more than half with increasing drought intensity (Figures 4C–E). Within water regimes, single AMF and PGPR inoculations mostly stimulated K, Ca, and Mg content significantly, but the dual AMF and PGPR inoculations always had a far greater effect of equal magnitude (Figures 4C–E).

In the most severe drought regime (FC30), the dual AMF or PGPR inoculation increased root N by 151%–138%, P by 176%–181%, K by 114%–112%, Ca by 126%–136%, and Mg by 130%–140% compared to non-inoculated seedlings (Figure 4).

### Foliar Nutrient Concentration

**Figure 5:**
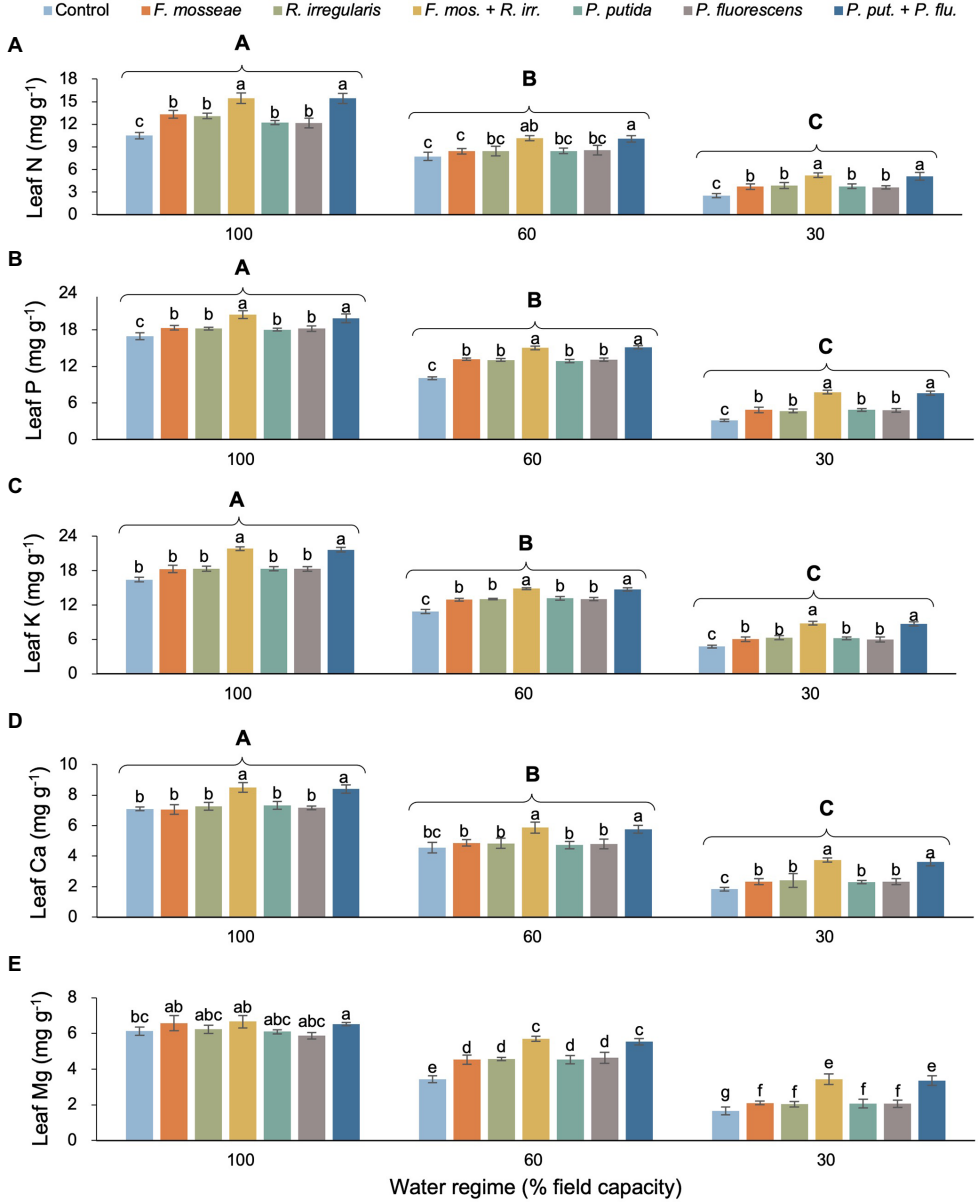
Effect of water regime and soil microbial inoculation on leaf N **(A)**, P **(B)**, K **(C)**, Ca **(D)**, and Mg **(E)** of *M. communis* seedlings (expressed on a dry weight basis). Water regime and inoculation treatment had significant main effects on all shown leaf nutrients but a water regime × inoculation treatment interaction only occurred for leaf Mg (Table 2). Therefore, different lower-case letters indicate significant differences between microbial inoculation treatments across water regimes for leaf Mg but only within water regimes in the remaining root nutrients (Duncan’s new multiple range test, *α* = 0.05). Different upper-case letters indicate significant differences between water regimes (Duncan’s new multiple range test, *α* = 0.05).

We found no evidence of a statistically significant interaction between water regime and microbial inoculation for any of the measured leaf nutrients apart from Mg (Table 2). However, as main effects, both water regime and microbial inoculation had a significant influence on foliar nutrient concentration. Regardless of the inoculation treatment, increasing water deficit reduced foliar N, P, K Ca, and Mg by at least about 50% (Figures 5A–E). In most cases, single AMF or PGPR inoculation caused a significant increase of similar magnitude in foliar nutrient status compared to non-inoculated controls (Figures 5A–E). However, peak foliar nutrient concentrations were invariably associated with dual AMF and PGPR inoculations (both with the same effect size) and the differences to singly inoculated seedlings and the non-inoculated control were almost always statistically significant (except for leaf N in the FC60 treatment and Mg in the FC100 treatment; Figures 5A–E). In the FC30 treatment, dual AMF and PGPR inoculations promoted N concentration by 102%–107%, P by 143%–149%, K by 83%–84%, Ca by 10%–98%, and Mg by 102%–106% compared to the non-inoculated control (Figures 5A–E).

## Discussion

*Myrtus communis* is an ecologically important species in its natural range and economically significant in (semi-)arid growing areas around the world. However, the rapidly changing climate complicates cultivation and, together with land use change and rampant illegal harvesting, imperils natural habitats (Amiri et al., 2015). In this study, we therefore tested whether AMF and PGPR inoculation may enhance the drought resistance of common myrtle to support habitat restoration programs in its native range and promote sustainable cultivation practices across the globe.

### Biomass Allocation

Foliage, stem, and root biomass decreased with increasing drought stress, but the inoculation with microorganisms, especially the dual AMF and PGPR inoculations fully or at least partially compensated for the negative effects of drought stress when compared to the uninoculated, well-watered control (Figure 1). A reduction in leaf area and thus leaf biomass provides an effective means to curtail water use and is a common adaptation to drought in many plant species (Larcher, 2003). Our companion study showed drought-induced reductions in foliar gas-exchange of the myrtle seedlings that were fully or partially compensated by dual AMF or PGPR inoculation in the FC60 and FC30 treatments, respectively (Azizi et al., 2021). Growth processes have been shown to respond more sensitively to abiotic stresses (such as drought) than photosynthesis and it is therefore likely that the growth reductions seen in the stems and roots of our myrtle seedlings are due to carbon sink limitation rather than a limited supply of photo-assimilates (Palacio et al., 2013). Moreover, lack of soil water at any plant growth stage reduces the absorption, transport, and metabolization of nutrients, impairing carbon storage and dry matter (Hu and Schmidhalter, 2005). A disruption of the root-soil interface, which can already occur under mild drought stress, results in substantial losses in root conductivity triggering stomatal closure prior to the onset of cavitation (Kumar et al., 2010; Rodriguez-Dominguez and Brodribb, 2020). However, AMF-induced changes in root morphology and the vast hyphal-driven expansion of the root system strongly increase root uptake and nutrient transport (Orfanoudakis et al., 2010). PGPR also effectively improve seed germination, accelerate growth in the early stages, induce root formation, and increase the number of root hairs (Heinonsalo et al., 2004). Some rhizobacteria contain the enzyme 1-aminocyclopropane-1-carboxylate (ACC) deaminase that reduces the production of ethylene, which in turn regulates auxin synthesis and allocation, ultimately leading to stimulation of root growth (Xie et al., 1996; Růžička et al., 2007). Both *Pseudomonas* species used in our study have been shown to produce ACC (Saravanakumar and Samiyappan, 2007; Gamalero et al., 2008) suggesting that the increase in root growth observed in our myrtle seedlings is related to ACC activity. Increasing biomass and growth of various crops (e.g., rice, foxtail millet, and peppermint) resulting from AMF and PGPR soil inoculation during drought conditions have also been reported in agreement with our findings (Ruíz-Sánchez et al., 2011; Gong et al., 2015; Chiappero et al., 2019).

### Water Relations

In line with the results reported for *Juglans regia* and *Eucaplytus camaldulensis*, WUE remained unaffected by soil microbial inoculation (Liu et al., 2019; Mateus et al., 2021), but increased with increasing drought intensity in our myrtle seedlings (Figure 2). Water deficit affects photosynthesis and transpiration to different degrees, resulting in significant differences in WUE (El Hafid et al., 1998). WUE usually rises under drought stress, but this increase is not associated with enhanced production because the increase is driven by reduced transpiration rather than an increase in photosynthetic carbon assimilation (Blum, 2009). In the present study, *g*_m_ decreased with increasing water deficit stress, probably linked to reduced leaf water status and thus tissue hydration (Azizi et al., 2021; Figure 3). Lower rates of photosynthetic CO_2_ assimilation in the presence of higher levels of *C*_i_ indicate low *g*_m_ and impaired carbon metabolism of mesophyll cells (Ratnayaka and Kincaid, 2005). Microbial soil inoculation, particularly dual AMF and PGPR applications, mitigated drought-related reductions in *g*_m_, which was mirrored by lower *C*_i_ values indicating greater utilization of absorbed CO_2_. Similar findings in terms of *C*_i_ and *g*_m_ were obtained with *Azospirillum brasilense* and *Bacillus* sp. in seedlings of the tropical canopy tree *Cariniana estrellensis* (Tiepo et al., 2018), and with *Rhizophagus irregularis* in seedlings of *Robinia pseudoacacia* (He et al., 2016) under drought conditions.

The RWC of *M. communis* seedlings decreased with increasing drought stress, but soil inoculation with AMF or PGPR (especially the dual inoculations) largely or fully compensated for these declines in the FC60 treatment (also compared to the well-watered and uninoculated control) and still had a positive, albeit less pronounced, effect in the FC30 treatment (Figure 3). A similar pattern was observed for leaf water potential of these myrtle seedlings published recently as part of the overarching study (Azizi et al., 2021). *Cupressus atlantica* and tropical tree seedlings also showed increases in leaf RWC in response to AMF or PGPR treatments (Zarik et al., 2016; Tiepo et al., 2018). AMF hyphal networks greatly expand the root surface thus giving plants access to a larger soil volume and because of their small diameter they can grow into the smallest pores and crevices that fine roots could not reach. The symbiosis with AMF allows plants to take up more water and thus maintain favorable water relations over a wider range of environmental conditions and under drought, AMF have been shown to be able to increase hyphal moisture uptake (Augé et al., 2015). PGPR have been demonstrated to improve the water relations of seedlings by producing phytohormones that favorably affect plant water relations such as abscisic acid and auxin (Kothari et al., 1990; Boiero et al., 2007).

### Root and Leaf Nutrient Concentration

Root and leaf N concentration decreased under water deficit conditions, but the soil inoculation treatments (especially dual AMF or PGPR inoculations) partially offset the negative drought effects on plant nutrient dynamics compared the non-inoculated control seedlings (Figure 5). These results are consistent with the studies of Abbaspour et al. (2012) on *Pistacia vera* seedlings and with those of Armada et al. (2015) on *Zea mays*. Rhizosphere bacteria increase the rate of nitrate translocation from root to shoot by increasing the amount of cytokinin in the host plant (Flores et al., 2005).

Mycorrhizal fungi also activate glutamine synthetase, arginase, and urease by affecting root physiology and thus increase the N uptake and utilization efficiency of host plants. Arginase and urease are key enzymes in the transfer of N from the mycelium to the roots of the host plant. Several ammonium and nitrate transporters have been identified in the mycelium of AMF (Tisserant et al., 2012) enabling mycelial N uptake in the form of nitrate or ammonium and subsequent conversion to organic compounds by glutamine synthetase (Bago et al., 2001).

In agreement with the findings of Zhang et al. (2019) and Ortiz et al. (2015), root and leaf P content in our myrtle seedlings decreased with increasing drought stress but AMF and PGPR inoculation mitigated this effect (Figure 5). While both single and dual inoculations produced significant positive effects in leaf P, root P responded more strongly to the dual inoculation treatments. This finding implies that multiple AMF or PGPR acting in concert are required to significantly increase P availability and subsequent root uptake while the activity of single AMF or PGPR species seems to be sufficient to steer allocation toward increased P supply to the leaves. Phosphorous plays an important role in many plant physiological processes linked to energy storage and transfer, photosynthesis, regulation of enzyme activity, and carbohydrate transport and it also affects plant water relations (Waraich et al., 2011). Numerous studies have shown that following AMF inoculation, P uptake in plants increases under stress conditions (Garg and Manchanda, 2008; Bowles et al., 2016), which has been attributed to the secretion of organic acids and phosphatase enzymes solubilizing inorganic P from soil minerals and mineralizing organic P sources (Subramanian et al., 2006).

Water deficit stress negatively affected root and leaf K of *M. communis* seedlings, but the inoculation treatments, especially the dual AMF and PGPR inoculations increased K uptake in both organs (Figure 5). Potassium is crucial for turgor control and thus for cell expansion during growth and guard cell osmoregulation (stomatal control), not to mention its key role in maintaining plasma membrane potential. The amount of plant-available K depends on the K content in the soil solution and the level of exchangeable K (Haby et al., 1990). Both K sources may increase in the presence of AMF and PGPR through organic acid-mediated silicate weathering and mineral dissolution causing the release of K thus allowing plants to increase their uptake (Campanelli et al., 2013).

Calcium plays a vital role in the regulation of many physiological processes in plants, thereby affecting growth processes and responses to environmental stresses. For example, the movement of water and dissolved mineral salts is affected by Ca through its influence on membrane structure and stomatal function, cell division, and cell wall construction (Hu and Schmidhalter, 2005). However, under water deficit stress, reduced water uptake and curtailed transpiration result in a decrease in leaf Ca content (Maksimović et al., 2003). In line with the findings of Dominguez-Nuñez et al. (2014) on *Pinus halepensis* and Meddich et al. (2015) on *Phoenix dactylifera*, we observed AMF- and PGPR-induced increases in root and foliar Ca contents of myrtle seedlings (Figure 5). Apart from increases in Ca availability resulting from the exploitation of a larger soil volume through AMF hyphae and accelerated mineral weathering linked to PGPR secretions, the positive inoculation effect on Ca levels in myrtle seedlings may in part be due to increases in stomatal conductance and transpiration given the role of xylem sap flow in Ca mobility (Ruiz-Lozano et al., 1995; Wu and Xia, 2006; Garg and Bhandari, 2016; Azizi et al., 2021).

A very similar pattern of drought-induced decrease was observed for Mg contents in roots and leaves of *Myrtus* seedlings exposed to drought (Figure 5). Under water deficit stress, single and dual inoculations with AMF or PGPR increased the Mg content in seedling roots and foliage which is consistent with the results of Danielsen and Polle (2014) on *Populus* × *canescens* and Armada et al. (2015) on *Zea mays*. The increase in Mg uptake is probably due to the hyphal expansion of the root system and consequently enhanced uptake of this element by the plant in the case of AMF and likely results from microbial solubilization of Mg-bearing carbonates and minerals in the PGPR treatments (Smith and Read, 2008; Evelin et al., 2012).

Our study revealed that, depending on drought intensity, AMF or PGPR inoculation can largely or at least partially offset the detrimental effects of drought on biomass production, water relations, and nutrient dynamics of *M. communis* seedlings. Especially the dual inoculations proved to be very potent suggesting even greater benefits from inoculations including multiple AMF and PGPR species. These findings motivate further research testing the effects of combined AMF and PGPR inoculations and linked to this, determining the composition of the microbial consortium that optimally supports myrtle health and performance. Most importantly though, our results highlight soil inoculations with beneficial microorganisms as a cost-effective, easy-to-use tool to promote drought resistance of myrtle. Such readily applicable approaches are urgently needed in support of conservation initiatives geared toward restoring natural myrtle populations and habitats. At the same time, such methods can be used to refine operational nursery practices to help future-proof myrtle cultivation.

#### Resource Identification Initiative

https://scicrunch.org/resolver/RRID:SCR_016479

## Data Availability Statement

The raw data supporting the conclusions of this article will be made available by the authors, without undue reservation.

## Author Contributions

SA conducted the experiments, helped devise the study design, and wrote the first draft with help of MT. MT, main supervisor of SA, devised the idea and study design and co-wrote the first draft. AA provided technical input and assistance. CA and LG provided editorial input. MB guided the analysis and writing process of earlier versions and wrote the final version of the manuscript. All authors contributed to the article and approved the submitted version.

## Funding

This study was fully funded by the Tarbiat Modares University, Iran.

## Conflict of Interest

The authors declare that the research was conducted in the absence of any commercial or financial relationships that could be construed as a potential conflict of interest.

## Publisher’s Note

All claims expressed in this article are solely those of the authors and do not necessarily represent those of their affiliated organizations, or those of the publisher, the editors and the reviewers. Any product that may be evaluated in this article, or claim that may be made by its manufacturer, is not guaranteed or endorsed by the publisher.
